# Report of Surgical Cases

**Published:** 1861-04

**Authors:** Geo. K. Amerman

**Affiliations:** One of the attending Surgeons to the City Hospital


### REPORT OF SURGICAL CASES.

BY GEO. K. AMERMAN, M. D.,

*One of the Attendiag Surgeons to the City Hospital.*

CASE I—*Compound Fracture of the Tibia, extending into the Knee Joint—Extensive Suppuration of the Joint—Recovery with a useful limb.*

M—— M——, aged 20, occupation a tinsmith; on the 19th of Nov., 1860, whilst engaged on the roof of a one story building, his foot slipped and he fell about twenty feet, striking, first on the right foot, and then, falling over on the same side. He was immediately carried out to an adjoining building and everything comfortably arranged by his fellow workmen until my arrival, about an hour after the occurrence of accident. I found his injuries confined to the right leg, with the exception of a scalp wound so trivial as not to require any attention. The injury of the leg consisted of a compound fracture of the tibia, oblique in its direction from below upwards, and before backwards, beginning, anteriorly, just below the tubercle, and terminating in the middle of each tuberosity above, so that without extending through the whole diameter of the bone, it formed a communication between the joint and an external irregular opening just below the tubercle. The cavity of the joint was filled with blood; the patella, separated from the condyles of the femur and drawn upward by the action of the quadriceps extensor. The wedge-shaped piece of the tibia, separated a considerable distance from the body of the bone and tilted forwards and upwards by the action of the ligamentum patella. The hemorrhage, quite free from a wound of one of the articular arteries, requiring considerable pressure over the wound to control it. A posterior straight splint was adjusted, with a roller, the patient placed in an express wagon and removed to his residence, a distance of three miles, before any dressings proper were applied. The first dressing employed was simply a fracture box, so arranged that free access could be obtained to the wound and the parts easily examined. The only tendency to displacement was a tilting forward of the fractured piece of the tibia, which proved very difficult to overcome. Severe constitutional reaction, with intense pain in the joint, supervened the day following the injury, which were met by full doses of opium internally, and the local application of the arnica and lead lotion.

Nov. 23—Pulse 100, skin hot, leg œdematous, joint immensely swollen and painful. Ordered a cathartic of Pulv. Seidlitz, and after its operation, to take Tr. Opii Camph., Spts. Nit. Dulc. aa ℥ i, Tr. Digitalis ʒ i, a teaspoonful every three hours. Continue the same local treatment.

Nov. 27—Constitutional excitement entirely subsided; pulse 90. On the 25th poultices were substituted for the arnica and lead lotion, since which time there have been a few discharges of unhealthy pus from the external wound. Pressure over the joint increases the discharge. The parts surrounding are œdematous and reddish, exhibiting a tendency to erysipelas. Continue the poultices, in place of iron mixture give Quin. et Ferri, full diet, and beer.

Dec. 10—The purulent discharge continued abundant, but of a more healthy character, until night before last, when a large abscess, situated five or six inches above the knee, on the outer side of the thigh, opened spontaneously, and discharged (according to the patient's statement) a quart of pus. Since then, the original opening on the tibia in front has ceased to discharge and is nearly closed. On introducing a probe into the opening above the knee, last formed, it readily passes into the cavity of the joint. The limb was now taken from the fracture box, a roller applied from the toes as far up as the seat of the fracture, a compress placed over the fragment, and the roller continued, applied lightly, as far as the upper part of the thigh. To this was added a posterior straight splint, extending from the middle of the thigh to the foot, secured by a second roller. The same constitutional treatment as last noted.

Jan. 4—Since last date the discharge has gradually diminished. The original wound has entirely closed. The opening above the knee still patent and discharging a small quantity of healthy pus. There is no pain except on moving the limb. The fragment can be easily moved, producing crepitus. The same dressings reapplied and same constitutional treatment.

Feb. 20—All dressings removed, and the patient allowed to walk about, with the use of a cane. The opening above the knee entirely closed. The joint free from pain or tenderness in any part and capable of supporting easily the weight of the body. Flexion can be carried to nearly a right angle and extension is almost perfect. Either flexion or extension carried beyond a certain point produce pain. With the exception of a moderate swelling around the joint, due to inflammatory exudation external to its cavity, and a slight elevation of the patella, it presents the same appearance as its fellow of the opposite side, and I have every reason to believe that by frictions and passive motion, persevered in for a long time, it will be quite as useful.

The only point of interest in connection with the above case is, its favorable termination. Simple fractures, complicated with injury of a large joint, are always regarded seriously, and compound fractures extending into a joint in such a way as to seriously damage so important a part, have, in times past, been regarded of so grave a character as to demand immediate amputation. This question of immediate amputation for injury is always a difficult one for the surgeon to decide, and must necessarily remain so. But as there is a difference of opinion in the profession regarding the results of primary and secondary amputations, with, it seems to me, the strongest evidence in favor of a secondary operation, I am inclined to the belief that in all cases of doubt we should attempt to preserve a member, and if we fail in this, then resort to an operation secondarily.

Case II—*Extirpation of the Eye for Injury—Recovery.*

Mrs. A——, aged 31, of a nervous temperament, but in the enjoyment of good general health, first came under my charge in July, 1860. On the 4th of July, 1859, a piece of percussion cap, from a small pocket pistol, struck her left eye ball near its centre, with such force as to lacerate the cornea, and, as was supposed, burrying itself deeply in the organ. Intense pain and almost entire loss of sight were an immediate result of the injury. Several attempts were made to extract the foreign body, but as it was uncertain in what direction it lay, or how deeply it had penetrated, or even whether it was lodged in the eye at all, they were unsatisfactory and entirely unsuccessful. Some inflammation supervened, accompanied by excruciating pain in the eye, forehead and side of the face. Leeches, blisters and collyria, without number, were resorted to without benefit. The inflammation pursued a regular course, to the complete destruction of the organ—the pain becoming after a time intermittent and so intense as to require the administration of large doses of opium to procure any relief whatever. In July, 1860, one year after the accident, she came under my care. At that time her general health was considerably impaired, the remains of the injured eye in a chronic state of inflammation, and the sound one exhibiting occasional symptoms of sympathetic action. I advised the immediate removal of the diseased eye as the only safeguard against destructive inflammatory action being excited in the other, and on the 20th of July, assisted by Dr. C. J. Taggart, of Beloit, and Dr. Chas. G. Smith, of this city, I performed the operation of Critchett for extirpation of the eye, the patient being under the influence of an anæsthetic. Simple dressings of cold water were applied for two days, and then all treatment discontinued. Not an unfavorable symptom occurred, and in two weeks the parts were in an excellent condition for an artificial eye. As regards the method of extirpation of the eye first recommended by Mr. Critchett, it is thus described in the January number of the *N. Y. Journal of Medicine* for 1859, by Dr. C. R. Agnew, Surgeon to the New York Eye Infirmary: "The first step is to act through the conjunctiva ocali by a circumsection of the cornea in a line with the insertion of the recti muscles; then to open the theca oculi close to the sclerotic implantation of the external rectus, and a little below its inferior border. All this may be done with a pair of toothed forceps and a pair of scissors, the latter so made as to cut by their very points, and yet so blunted as not to endanger the sclerotic. The sclerotic is easily recognized by its glistening non-vascular appearance; and, moreover, immediately upon opening the theca oculi, a watery fluid exudes. Having exposed the lower border of the tendon of the rectus, an ordinary strabismus hook is slipped under the muscle and a cut or two of the scissors soon severs it from its sclerotic implantation. Care should be taken to cut between the hook and the cornea, in such a manner as to leave as little of the muscle as possible attached to the eye-ball. Having divided one rectus the other steps are easy. The strabismus hook when pushed in a line of the first section, naturally glides beneath the theca oculi and the next rectus. It is better to divide the superior rectus after the external, and then the inferior rectus, leaving the internal for the closing sweep of the scissors after the optic nerve has been severed. After dividing the muscles the cavity of the ocular sheath is fairly invaded and nothing remains but to divide the optic nerve and internal rectus. The division of the optic nerve is first effected by means of the straight, blunt scissors. They should be gently pushed between the temporal aspect of the theca oculi and the sclerotic, until their closed points impinge upon the optic nerve at its junction with the eye-ball. A gentle rocking motion and a slight separation of the blades will cause them to enclose the nerve-trunk, which may be cut at a single clip. When the optic nerve is not shrivelled by atrophy its section communicates a decided jarring sensation to the operator's hand. As soon as the nerve is divided the eye-ball bulges forwards and may be easily tilted upon the side of the nose and the internal rectus divided. The exact moment of the section of the oblique muscles varies, depending upon the precision with which the scleroatic implantations of the recti are sought for. The section of the optic nerve is followd by a welling up of blood, which invariably ceases without requiring ligatures, a few moments after the eye-lids fall into position. And I would recommend that no attempts be made to free the sub-palpebral space from blood; the wounded parts will adjust themselves in such a manner as to control the hemorrhage."

Regarding the propriety of this operation Dr. Agnew says, in the same article above referred to, "There is no principle of practice in ophthalmic surgery so incontrovertible as that of the propriety of extirpating a diseased eye, with the aim of stopping sympathetic trouble in the fellow organ."

